# Case Report: Upfront sotatercept-based triple therapy in high-risk systemic sclerosis-associated pulmonary arterial hypertension

**DOI:** 10.3389/fcvm.2026.1956123

**Published:** 2026-10-07

**Authors:** Yuichi Tamura, Sarasa Isobe, Hiroyuki Fujii, Yuichiro Shirai, Masataka Kuwana

**Affiliations:** 1 Pulmonary Hypertension Center, International University of Health and Welfare Mita Hospital, Tokyo, Japan; 2 Department of Allergy and Rheumatology, Nippon Medical School Graduate School of Medicine, Tokyo, Japan

**Keywords:** case report, pulmonary arterial hypertension, risk stratification, sotatercept, systemic sclerosis, upfront combination therapy

## Abstract

Systemic sclerosis-associated pulmonary arterial hypertension (SSc-PAH) carries a poor prognosis, and treatment decisions are often complicated by coexisting left-heart disease, lung disease, chronic thromboembolic disease, or pulmonary veno-occlusive disease-like vasculopathy. The role of sotatercept as part of initial therapy in treatment-naive SSc-PAH has not been established. We report a 60-year-old man with diffuse cutaneous systemic sclerosis, first recognized at referral 3 years after the onset of skin symptoms, and high-risk precapillary PAH. Careful evaluation showed no clinically significant parenchymal lung disease, chronic thromboembolic disease, or post-capillary physiology. Upfront therapy was started with macitentan, tadalafil, and sotatercept, with short-term dobutamine support for low-output hemodynamics. After 3 months, right heart catheterization showed marked improvement: pulmonary vascular resistance decreased from 12.6 to 3.7 Wood units, mean pulmonary arterial pressure decreased from 43 to 30 mmHg, and cardiac index increased from 1.50 to 2.90 L/min/m². World Health Organization functional class improved from III to II, and risk status shifted to low risk by REVEAL Lite 2, COMPERA 2.0, and the Japanese Circulation Society/Japanese Pulmonary Circulation and Pulmonary Hypertension Society 2025 model. No sotatercept-related adverse events were observed. This case documents the short-term course of upfront sotatercept-based triple therapy in carefully selected high-risk precapillary SSc-PAH.

## Introduction

Systemic sclerosis-associated pulmonary arterial hypertension (SSc-PAH) is a severe connective tissue disease-associated PAH phenotype with poorer outcomes than idiopathic PAH and frequent competing contributors to pulmonary hypertension (1). In treatment-naive high-risk PAH, current guidelines support rapid risk-driven escalation, with parenteral prostacyclin-based triple therapy remaining the conventional guideline-supported approach (2, 3). Initial oral combination therapy with an endothelin receptor antagonist (ERA) and a phosphodiesterase type 5 inhibitor (PDE5i) is supported in connective tissue disease-associated PAH, particularly in low- or intermediate-risk patients (4).

Sotatercept, an activin signaling inhibitor, improved exercise capacity and clinical outcomes as add-on therapy in established PAH, high-risk PAH receiving maximally tolerated background therapy, and PAH diagnosed within the preceding year (5–7). However, these trials did not evaluate sotatercept as part of an upfront regimen in treatment-naive patients. A recent case described sotatercept-based initial triple therapy in severe idiopathic PAH (8). We report a patient with newly diagnosed high-risk SSc-PAH who achieved low-risk status within 3 months after upfront treatment with macitentan, tadalafil, and sotatercept.

## Case description

### Patient information

A 60-year-old man was referred for progressive exertional dyspnea and leg edema. Three years before referral, he first noticed tightness and discomfort of the skin of the forearms and face. Raynaud phenomenon developed approximately 1 year before referral, at about the same time as the onset of exertional dyspnea. Systemic sclerosis had not been diagnosed before referral, and he had therefore never been included in a systematic PAH screening program. Pulmonary hypertension was suspected at the referring hospital 1 month before referral on the basis of echocardiography performed for these symptoms, and systemic sclerosis and PAH were diagnosed concurrently at our center. He had marked coronary artery calcification and previous percutaneous coronary intervention to the left circumflex artery.

### Clinical findings

At the initial evaluation, skin thickening involved the face, forearms, and lower legs, with partial involvement of the chest and abdomen as well as sclerodactyly of the fingers, whereas the upper arms showed atrophic skin with hyperpigmentation, indicating previously more extensive involvement. The modified Rodnan skin score was 17 (maximum 51). Numerous patchy telangiectasias were prominent over the face (Figure 1), whereas digital ulcers and pitting scars were absent. Nailfold capillaroscopy showed an active scleroderma pattern. The clinical findings fulfilled the classification criteria for systemic sclerosis. Involvement of the trunk and of the upper arms, together with an interval of only approximately 2 years between the first skin symptoms and the onset of Raynaud phenomenon, indicated diffuse cutaneous disease; the atrophic upper-arm skin and the 3-year interval from the first non-Raynaud manifestation indicated that the peak of skin involvement had already passed at the time of referral.

**Figure 1:**
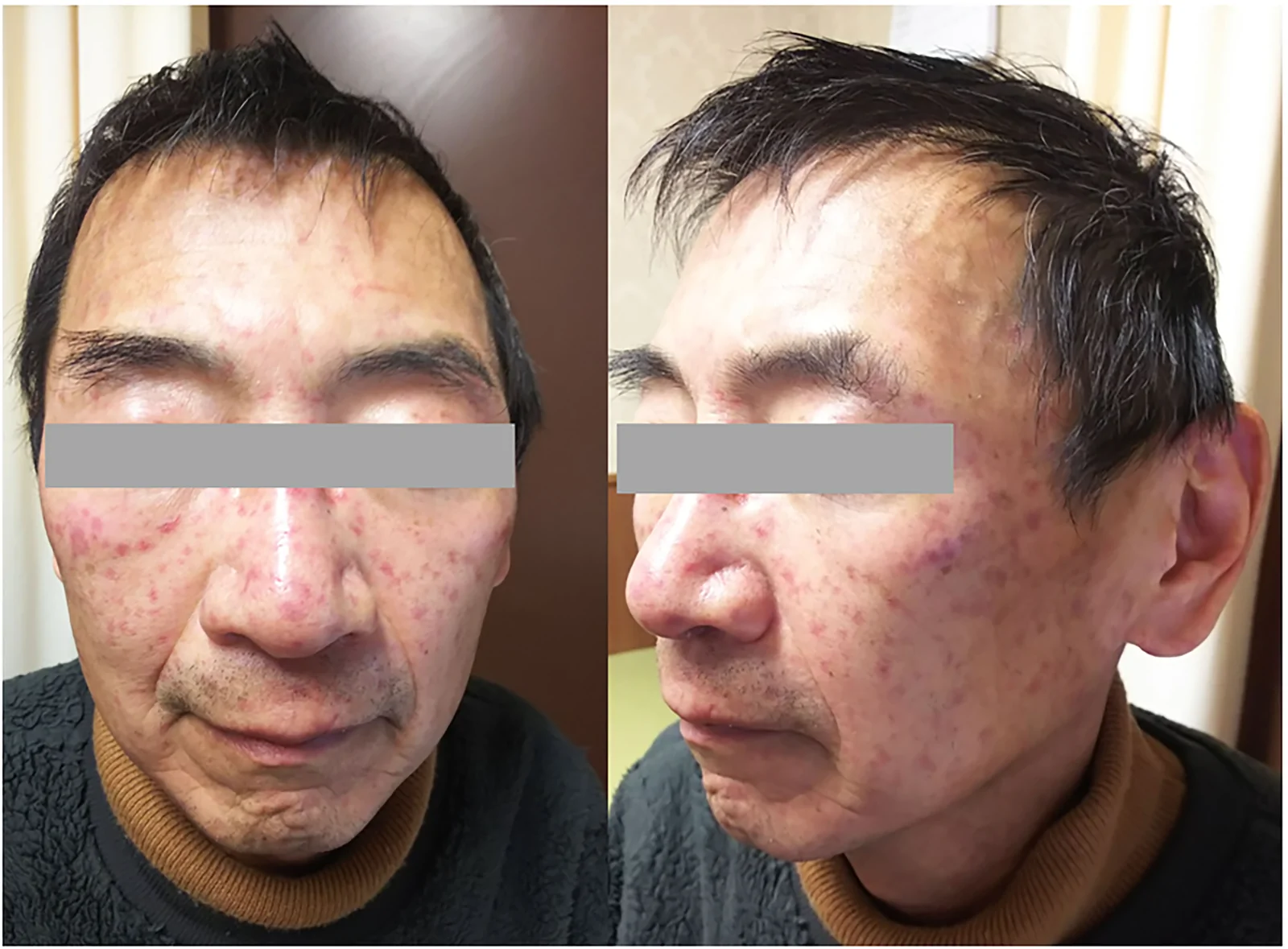
Facial telangiectasias in a patient with systemic sclerosis. Anterior (left) and left oblique (right) views of the patient's face. The patient exhibits numerous disseminated reddish-purple, macular telangiectasias across the face, particularly prominent on the cheeks, nose, and forehead. These findings are characteristic of the patient's underlying diffuse cutaneous systemic sclerosis. The eyes are obscured for privacy.

Antinuclear antibody testing showed both speckled and nucleolar patterns. Among the autoantibodies available in routine clinical practice, anticentromere, anti-topoisomerase I, and anti-RNA polymerase III antibodies were negative. The nucleolar pattern raises the possibility of an SSc-specific antibody that is not measurable in routine practice, such as anti-U3RNP or anti-Th/To antibody, both of which are associated with PAH. There was no evidence of overlap with other connective tissue diseases, including systemic lupus erythematosus, idiopathic inflammatory myopathy, or rheumatoid arthritis.

### Diagnostic assessment

The main diagnostic challenge was to distinguish group 1 SSc-PAH from other potential contributors to pulmonary hypertension in systemic sclerosis. Pulmonary function testing showed no impairment of lung function associated with decreased %DLCO. High-resolution computed tomography showed minimal subpleural fibrosis without honeycombing, and no findings suggestive of pulmonary veno-occlusive disease-like vasculopathy, such as smooth interlobular septal thickening, centrilobular ground-glass opacity, or mediastinal lymphadenopathy. Lung perfusion scintigraphy showed no segmental perfusion defects, and clinically significant parenchymal lung disease was not evident. Despite his coronary history, pulmonary arterial wedge pressure was 9 mmHg and left ventricular function was preserved, arguing against post-capillary pulmonary hypertension.

Right heart catheterization confirmed precapillary pulmonary hypertension: mean pulmonary arterial pressure 43 mmHg, pulmonary arterial wedge pressure 9 mmHg, cardiac output 2.69 L/min, cardiac index 1.50 L/min/m², and pulmonary vascular resistance 12.6 Wood units. He was in World Health Organization functional class (WHO-FC) III. Baseline risk assessment classified him as high risk by REVEAL Lite 2 (score 9), intermediate-high risk by COMPERA 2.0 four-strata assessment, and high risk by the Japanese Circulation Society/Japanese Pulmonary Circulation and Pulmonary Hypertension Society (JCS/JPCPHS) 2025 model (3). The JCS/JPCPHS model defines low-risk parameters as WHO-FC I-II, 6-minute walk distance >440 m, right atrial pressure <8 mmHg, cardiac index ≥2.5 L/min/m², and mean pulmonary arterial pressure ≤40 mmHg; 0–2 low-risk parameters indicate high risk, 3 indicate intermediate risk, and 4–5 indicate low risk (3). This patient fulfilled only 1 of 5 low-risk criteria at diagnosis. The diagnostic and treatment timeline is summarized in Figure 2.

**Figure 2:**
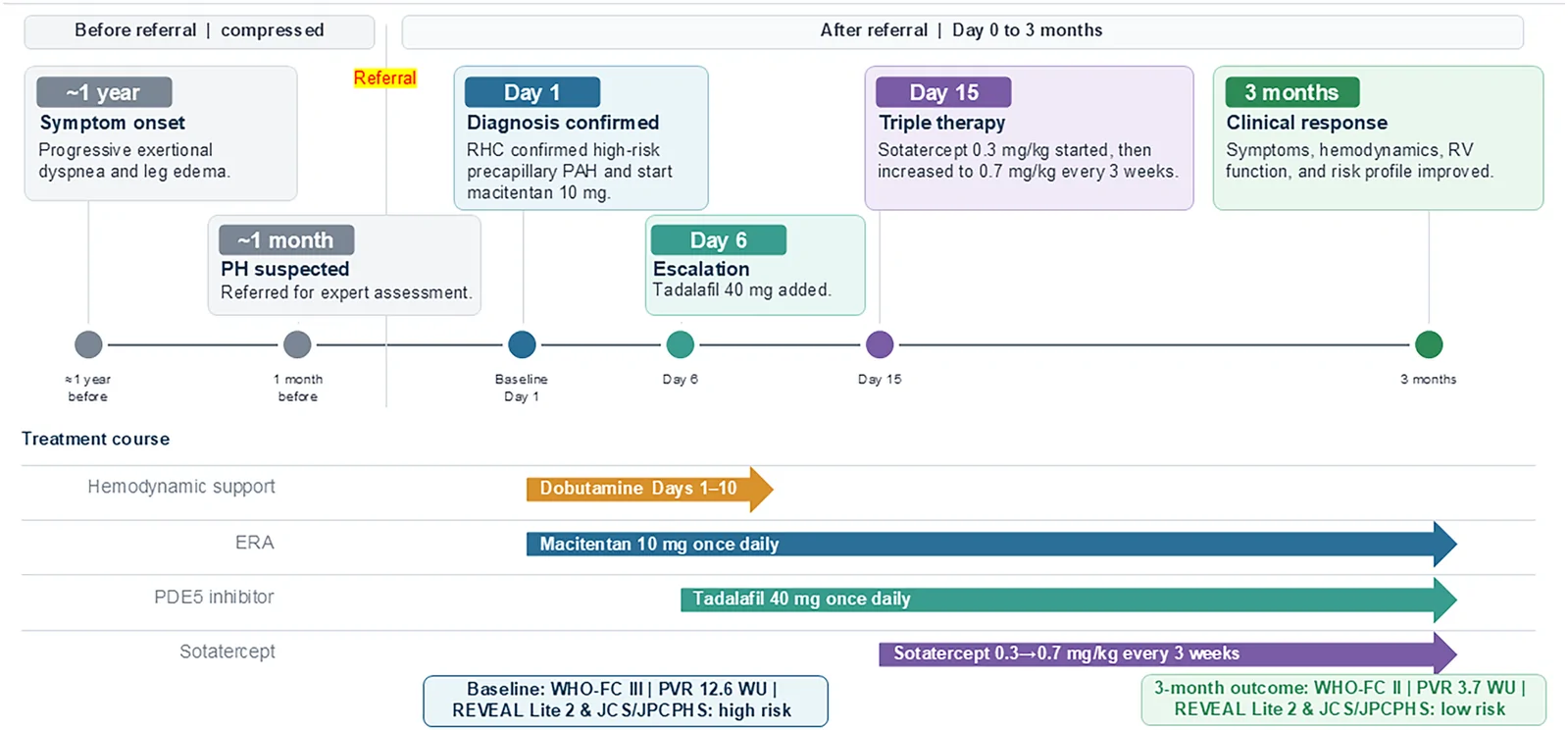
Clinical course and therapeutic escalation. Timeline of diagnosis, initial hemodynamic support, upfront combination therapy, and 3-month response. Timeline is compressed before referral and is not drawn to scale. RHC, right heart catheterization; PAH, pulmonary arterial hypertension; PH, pulmonary hypertension; ERA, endothelin receptor antagonist; PDE5, phosphodiesterase type 5; RV, right ventricular; WHO-FC, World Health Organization functional class; PVR, pulmonary vascular resistance; WU, Wood units; JCS/JPCPHS risk classification: low risk at 3 months.

### Therapeutic intervention

Treatment was started immediately after diagnosis. Macitentan 10 mg once daily was initiated on day 1. Because of low-output hemodynamics, dobutamine was administered from days 1 to 10 for hemodynamic support on initial therapies. Tadalafil 40 mg once daily was added on day 6. Sotatercept was initiated on day 15 at 0.3 mg/kg, increased to 0.7 mg/kg, and continued every 3 weeks as maintenance therapy.

Initial triple therapy including intravenous or subcutaneous prostacyclin, as recommended for high-risk patients at diagnosis, was considered first. Several patient-specific factors led us not to select this option. Firstly, skin sclerosis involving the forearms, lower legs, and abdomen, together with atrophic skin changes of the upper arms, limited suitable subcutaneous infusion sites and raised concern regarding infusion-site pain, impaired absorption, and skin breakdown. Secondly, the indwelling central venous catheter required for intravenous epoprostenol carries a risk of catheter-related bloodstream infection, a particular concern in systemic sclerosis. Additionally, after a full explanation of the guideline-recommended options, including intravenous epoprostenol, the patient declined parenteral therapy.

### Follow-up and outcomes

At 3 months, WHO-FC had improved from III to II and leg edema had resolved. Repeat right heart catheterization showed marked hemodynamic improvement (Table 1). Pulmonary vascular resistance decreased by approximately 71%, and cardiac index increased from 1.50 to 2.90 L/min/m². Echocardiography showed improved right ventricular function. Risk status improved to low risk by REVEAL Lite 2 (score 4), COMPERA 2.0, and the JCS/JPCPHS 2025 model. For the JCS/JPCPHS model, the number of low-risk parameters increased from 1 of 5 at baseline to 4 of 5 at 3 months. No adverse events considered related to sotatercept were observed during follow-up. In particular, there was no bleeding, thrombocytopenia, symptomatic hemoglobin rise, worsening facial telangiectasia, or worsening hypoxemia.

**Table 1:** Clinical, hemodynamic, and risk-profile changes.

Variable	Baseline	3 months	Change
Right heart catheterization			
Right atrial pressure, mmHg	5	3	−2
Mean pulmonary arterial pressure, mmHg	43	30	−13
Pulmonary arterial wedge pressure, mmHg	9	11	+2
Cardiac output, L/min	2.69	5.21	+2.52
Cardiac index, L/min/m²	1.50	2.90	+1.40
Pulmonary vascular resistance, Wood units	12.6	3.7	−8.9
Mixed venous oxygen saturation, %	63.4	75.3	+11.9
Echocardiography			
TAPSE, mm	18	20	+2
TAPSE/sPAP, mm/mmHg	0.20	0.34	+0.14
Right ventricular fractional area change, %	6	21	+15
Tricuspid regurgitation	Moderate	Mild	Improved
Pericardial effusion	Mild	None	Resolved
Other parameters			
WHO-FC	III	II	Improved
6-minute walk distance, m	351	393	+42
BNP, ng/L	209	48	−161
Hemoglobin, g/dL	11.5	12.9	+1.4
Platelet count, ×10⁹/L	163	179	+16
REVEAL Lite 2	9, high risk	4, low risk	Improved
COMPERA 2.0 four-strata model	Intermediate-high risk	Low risk	Improved
JCS/JPCPHS 2025 low-risk criteria fulfilled	1/5	4/5	Improved
JCS/JPCPHS 2025 risk category	High risk	Low risk	Improved

BNP, B-type natriuretic peptide; COMPERA, Comparative, Prospective Registry of Newly Initiated Therapies for Pulmonary Hypertension; JCS/JPCPHS, Japanese Circulation Society/Japanese Pulmonary Circulation and Pulmonary Hypertension Society; PVR, pulmonary vascular resistance; sPAP, systolic pulmonary arterial pressure; TAPSE, tricuspid annular plane systolic excursion; WHO-FC, World Health Organization functional class.

## Discussion

This case demonstrated marked hemodynamic improvement and risk reduction over a short period after upfront macitentan, tadalafil, and sotatercept therapy with early inotropic support. The improvement cannot be attributed to sotatercept alone because all interventions were instituted during the same initial treatment period. Its reporting value therefore lies less in demonstrating efficacy than in documenting the rationale, short-term clinical course, and safety observations of an upfront sotatercept-containing regimen in a clinical situation for which direct trial evidence is lacking. Patients with connective tissue disease-associated PAH were included in the phase 3 sotatercept program, accounting for approximately 15% of participants in STELLAR and 30% in HYPERION. In all of these trials, however, sotatercept was added to established background PAH therapy: stable double or triple therapy in STELLAR, maximally tolerated background therapy in ZENITH, and background therapy in patients diagnosed within the preceding year in HYPERION (5–7). An initial, sotatercept-containing regimen in a treatment-naive patient with SSc-PAH has therefore not been evaluated. It is this treatment strategy, rather than the SSc-PAH population itself, that is not represented in the existing evidence base.

The rationale for considering early sotatercept in this patient was based on careful phenotyping. SSc-PAH is frequently complicated by potential contributors to pulmonary hypertension, including left-heart disease, parenchymal lung disease, chronic thromboembolic disease, and pulmonary veno-occlusive disease-like vasculopathy. These factors raise concern that intensification of PAH-directed therapy may be ineffective or may lead to unfavorable events, such as pulmonary congestion or worsening oxygenation, if the patient is misclassified (9). In the present case, lung perfusion scintigraphy, pulmonary evaluation, and hemodynamic assessment did not suggest chronic thromboembolic disease, clinically significant lung disease, or post-capillary physiology. Thus, despite the systemic sclerosis background, the patient fulfilled a hemodynamic and clinical phenotype consistent with group 1 PAH.

The JCS/JPCPHS 2025 guideline is particularly relevant to this case because it provides a published 3-strata risk model for Japanese practice and recommends applying the same treatment algorithm used for idiopathic PAH to SSc-PAH (3). In that guideline, high-risk patients at diagnosis are recommended to receive triple combination therapy consisting of intravenous or subcutaneous prostacyclin, an ERA, and a PDE5i, and activin signaling inhibition is positioned as add-on therapy for intermediate- or high-risk patients during follow-up after careful evaluation of prostacyclin therapy in high-risk cases (3). The present case should therefore not be interpreted as evidence to replace parenteral prostacyclin in high-risk PAH. However, as subcutaneous or intravenous therapies are sometimes avoided in patients with connective tissue diseases due to risks such as infections, the present case may provide useful information for building evidence aimed at modifying future treatment algorithms.

Available sotatercept trial data support the biological plausibility of this approach but do not establish the efficacy or safety of upfront use. In published trial reports and prespecified subgroup analyses, treatment effects and safety findings have not shown a clearly divergent pattern in connective tissue disease-associated PAH compared with the overall PAH population (5–7). At the same time, the transition to low-risk status and the marked hemodynamic improvement in this case are consistent with the emerging discussion that future PAH treatment goals may extend beyond risk reduction toward long-term disease control or remission (10).

The main limitation is that this is a single case with short follow-up, concomitant initiation of multiple therapies, and no ability to isolate the effect of sotatercept. Additional real-world reports and prospective studies are required to determine whether upfront sotatercept-containing therapy can be translated into a safe and effective approach, and to define patient selection, timing of initiation, safety monitoring, and long-term outcomes in SSc-PAH.

The take-away lesson is that early sotatercept use may be clinically considered only after rigorous phenotyping confirms a precapillary group 1 PAH profile and after the limitations of guideline-supported parenteral prostacyclin alternatives have been explicitly weighed.

## Patient perspective

The patient provided written informed consent for publication. A separate patient-authored perspective statement was not available at the time of manuscript preparation.

## Data Availability

The clinical data supporting this case report are included in the article. Further inquiries can be directed to the corresponding author.
